# Gut Fungi in Alzheimer's Disease: Mechanisms, Biomarkers and Therapeutic Potential

**DOI:** 10.14336/AD.2024.1310

**Published:** 2026-05-06

**Authors:** Ziyu Liu, Zhuo Chen, Xiaoyan Zhang, Yi Ge, Duo Yuan, Huihong Zhai

**Affiliations:** Department of Gastroenterology, Xuanwu Hospital, Capital Medical University, Beijing 100053, China.

**Keywords:** Alzheimer's disease, intestinal fungi, gut microbiome, fecal microbiota, amyloid seeding hypothesis

## Abstract

Alzheimer’s disease (AD) is a progressive neurodegenerative disorder of aging that imposes a heavy medical and socioeconomic burden. Its multifactorial pathology—including amyloid-β (Aβ) accumulation, tauopathy, and chronic neuroinflammation—lacks effective disease-modifying treatments. Recent studies highlight the gut–brain axis, specifically the intestinal mycobiome (fungal community), as a novel factor in AD. In this review, we summarize evidence on gut fungi in AD. Altered gut fungal profiles have been reported in AD patients, including enrichment of *Candida tropicalis* and *Schizophyllum commune* and reduction of *Rhodotorula mucilagino*sa, and in AD mouse models, such as increased abundance of the *Dipodascaceae* family. Fungi can translocate or release bioactive molecules that impact the brain; for instance, fungal proteins (enolase, β-tubulin) and polysaccharides (chitin) have been detected in AD brain tissue. Fungal metabolites also emerge as potential biomarkers; notably, plasma sterigmatocystin levels were significantly higher in AD patients compared to controls. Mechanistically, gut fungi (such as Candida or Malassezia species) may activate microglia and promote Aβ deposition via inflammatory pathways, while fungal prion-like proteins can accelerate AD protein aggregation in vitro. Conversely, certain fungi exert neuroprotective effects; oral administration of the probiotic yeast *Saccharomyces boulardii* attenuated cognitive deficits and Aβ pathology in APP/PS1 mice. Importantly, fecal fungal profiling is non-invasive and may serve as a practical AD screening tool. Collectively, these findings nominate gut fungi as potential biomarkers and therapeutic targets. Future work should validate specific mycobiome signatures and develop fungus-targeted interventions to enable earlier diagnosis and novel treatments for AD.

## 1. Introduction

The global aging population is leading to a surge in dementia cases, with Alzheimer's disease (AD) identified as a major cause of progressive dementia. As of 2019, approximately 47 million individuals worldwide were afflicted with dementia, a number expected to escalate to around 131 million by 2050 [1]. The global incidence of male dementia is expected to be 0.5% among individuals aged 40-69, 6.5% among those aged 70-84, and 23.5% among those aged 85 and above by 2050. Similarly, the expected incidence of dementia among women is projected to be 0.6% for those aged 40-69, 8.5% for those aged 70-84, and 30.5% for those aged 85 and above [2]. Consequently, the economic impact of dementia is profound. In 2019, the estimated total global social cost of dementia was 1.3 trillion US dollars, equivalent to 1.5% of the global gross domestic product (GDP). It is anticipated that by 2030, this cost will more than double, reaching $2.8 trillion [3]. However, at present, treatment options for AD are limited primarily to symptomatic relief, and the effect is limited. Except for cholinesterase inhibitors and Memantine, as no other FDA-approved medications exist to enhance cognitive function in dementia patients [4]. Recent studies highlight the potential role of the gut microbiome of the central nervous system through the "microbiome-gut-brain axis". At present, there are articles confirming its intricate link to AD [5, 6]. This connection involves participation in the pathogenic mechanisms of AD, including Aβ protein deposition [7], tau protein hyper-phosphorylation, neuroinflammation [8, 9], oxidative stress injury [10, 11], and increased blood-brain barrier permeability [12].

Moreover, although the density of fungi in the gut is significantly lower than that of bacteria—leading to historically limited attention to the gut mycobiome—fungi still play important biological roles. The number of culturable fungi is estimated to range from 10^2^ to 10⁶ CFU/g, whereas bacterial densities can reach as high as 10¹¹–10¹^2^ CFU/g [13, 14]. Fungal genes account for only approximately 0.1% of the total microbial metagenome [15]. Nevertheless, intestinal fungi exert critical biological effects through their unique metabolic products, such as mycotoxins and immunomodulatory molecules. These fungal-derived compounds can profoundly influence both local and systemic immune responses, contribute to the regulation of mucosal barrier integrity, and activate inflammatory signaling pathways [16, 17].

Furthermore, a complex network of interactions exists among fungi, bacteria, and viruses within the gut microbiota. Emerging evidence indicates significant positive correlations between intestinal fungi and various bacterial genera. These mutualistic relationships are believed to be mediated through several mechanisms, including metabolic complementation, cooperative biofilm formation, quorum sensing signaling, and fungal regulation of intestinal oxygen levels. For instance, *Saccharomyces cerevisiae* can secrete amino acids that enhance the growth of *Lactobacillus* species, while *Candida* species have been shown to promote the proliferation of *Clostridium difficile* under anaerobic conditions [18]. At the same time, antibiotic-mediated depletion of bacterial populations has been shown to induce fungal dysbiosis, whereas antifungal interventions may likewise disturb the composition of bacterial microbiota [19], furthermore, in patients with mild cognitive impairment (MCI), significant co-occurrence relationships have been identified between specific fungal genera (such as *Wallemia* and *Cyberlindnera*) and bacterial genera (such as *Lachnospira* and *Bifidobacterium*). These observations suggest that gut fungi may indirectly influence host neurological function by modulating the overall microbial ecosystem.

Given the distinct fungal alterations observed in AD, gut fungi may hold potential as novel biomarkers for early diagnosis and disease monitoring [20]. With the continuous advancement of high-throughput sequencing, metabolomics, and proteomics technologies, mycobiome research is increasingly becoming an important direction for exploring the pathogenesis of neurodegenerative diseases such as AD and developing early intervention strategies. Therefore, this review focuses on the characteristics of intestinal fungi in patients with AD, the mechanisms by which fungi affect AD, and how fungi affect the treatment of AD. Understanding gut fungi's contribution to AD pathogenesis may reveal new strategies for therapeutic interventions and help mitigate the growing burden of dementia on society.

## 2. The strict connection between Intestinal Fungi and AD

Recent research has established a substantial connection between neurological diseases and intestinal fungi [21], Dysregulation of intestinal fungi has been documented in AD patients and mice. For instance, *Candida albicans* (NCYC 3115) has been shown to migrate from the gut to the brain, triggering inflammatory responses [22]. Furthermore, studies have found that fungal antigens and fungal polysaccharide can be detected in the blood serum of AD patients [23], meanwhile, the following fungi exist in the brain of AD patients, respectively: *Saccharomyces cerevisiae*, *Malassezia globosa*, *Malassezia restricta*, *Penicillium* and *Phoma spp* [24], highlighting the potential role of fungal colonization as a contributing factor to AD [25]. Additionally, studies have noted a significant reduction of the *Coprococcus* and an increased abundance of *Escherichia Shigella* and *Barnesiella* genera in the 3xTg-AD mice, indicating a pro-inflammatory state conducive to AD pathogenesis. Moreover, the fungal *Dipodascaceae* family was significantly increased, suggesting its involvement in AD's metabolic disturbances [26]. In AD patients, studies have also found that several key differential fungi such as *Candida tropicalis* and *Schizophyllum* commune were enriched in the AD patients, while *Rhodotorula mucilaginosa* decreased significantly [27]. Furthermore, since existing literature has already established a connection between intestinal bacteria and the progression of AD, and considering that some fungi, such as *Candida albicans*, interact with intestinal bacteria [28], it is reasonable to speculate that intestinal fungi could influence the progression of AD through their interaction with intestinal bacteria. In conclusion, cumulative evidence robustly supports a significant link of a strong association between intestinal fungi and the occurrence and development of AD. Underscoring the need for further research to elucidate intestinal fungi's role in AD pathogenesis and potential therapeutic interventions.

## 3. Fungal Characteristics in AD

Recent findings indicate that certain fungal species can migrate from the gut to the brain [22] in both AD (AD) patients and mouse models.. This migration not only alters the profile of intestinal fungi but also results in characteristic fungal infections within the brain. Therefore, the fungal characteristics in AD patients and AD mouse models will be described in the following aspects: the characteristics of intestinal fungi in AD patients and AD mouse models and the characteristics of fungal infections in the brain.

### 3.1 Characteristics of intestinal fungi in MCI patients and AD mouse models

Research into the intestinal fungi of AD patients is scarce. However mild cognitive impairment (MCI), a cognitive stage that often precedes AD [29-32], offers insights into early fungal changes. Therefore, the characteristics of intestinal fungi in patients with MCI are also of great significance for studying the characteristics of intestinal fungi in patients with AD. A study involving 17 participants showed no significant variance in the overall diversity of intestinal fungal communities between MCI patients and healthy controls. Nonetheless, MCI patients exhibited a slight decrease in fungal species diversity, characterized by a reduction in Ascomycetes and an increase in Basidiomycetes. Further analysis at the level of major fungi genera showed that the proportion of fungi in *Sclerotinia, Phaffomyceteceae, Trichocomaceae, Cystofilobasidiaceae, Togniniaceae, Botrytis, Kazachstania, Phaeoacremonium* and *Cladosporium* genera was significantly higher in MCI patients than in CN subjects, while the proportion of fungi in *Cladosporiaceae* and *Meyerozyma* genera was lower [21]. Moreover, an investigation of 88 AD patients from China identified altered fecal fungal microbiota composition, despite unchanged diversity. And several key differential fungi such as *Candida tropicalis* and *Schizophyllum* commune were enriched in the AD patients, while *Rhodotorula mucilaginosa* decreased significantly [27]. Another study found no significant difference in the diversity of intestinal fungi among the 3xTg-AD mice, one of the most widely used AD models. In specific fungal species, only the fungal *Dipodascaceae* family was significantly increased. This may be related to metabolic changes in AD [26] (Table 1).

**Table 1.** Fungi involved in AD or MCI.

Fungi	Disease	Research object	Alteration	Clinical significance
***Sclerotiniaceae***	MCI [21]	Human	↑in MCI	unknown
***Phaffomyceteceae***	MCI [21]	Human	↑in MCI	unknown
***Trichocomaceae***	MCI [21]	Human	↑in MCI	unknown
***Cystofilobasidiaceae***	MCI [21]	Human	↑in MCI	unknown
***Togniniaceae***	MCI [21]	Human	↑in MCI	unknown
***Botrytis***	MCI [21]	Human	↑in MCI	unknown
***Kazachstania***	MCI [21]	Human	↑in MCI	unknown
***Phaeoacremonium***	MCI [21]	Human	↑in MCI	unknown
***Cladosporium***	MCI [21]	Human	↑in MCI	unknown
***Cladosporiaceae***	MCI [21]	Human	↓in MCI	unknown
***Meyerozyma***	MCI [21]	Human	↓in MCI	*Meyerozyma* correlates significantly positively with Aβ42 and tau in CN subjects and correlates significantly positively with Aβ42 in MCI subjects.
***Candida tropicalis***	AD [27]	Human	↑in AD	The enriched abundance of *C. tropicalis* in AD correlated negatively with levels of IL-8 and IFN-γ, but correlated positively with levels of IP-10 and TNF-α
***Schizophyllum commune***	AD [27]	Human	↑in AD	*S. commune*, was positively correlated with levels of TNF-α and IP-10
***Rhodotorula mucilaginosa***	AD [27]	Human	↓in AD	*R. mucilaginosa* was negatively correlated with the level of TNF-α
***Dipodascaceae***	AD [26]	Mouse	↑in AD	Participate to metabolic and proinflammatory imbalances

↑, increase; ↓, decrease, AD, Alzheimer’s disease; MCI, mild cognitive impairment

These observations underscore the potential of intestinal fungi as biomarkers for AD. Given that gut microbiome changes can precede AD symptoms [33], integrating fungal biomarkers with current AD risk models could enhance early diagnosis and intervention strategies. At present, some papers have proposed models for predicting the risk of AD [34-36]. Therefore, in the future, it is possible to construct a more accurate AD risk prediction model by combining intestinal fungal biomarkers with existing AD risk prediction models, so as to realize early diagnosis and treatment of AD.

### 3.2 Characteristics of fungal infection in the brain of patients with AD

Research has identified fungal components in postmortem brain tissue of Alzheimer's patients, including peptides closely linked to fungal proteins [24], as well as a variety of fungal DNA [37]. In addition, fungal macromolecules such as glucans, proteins, and DNA have been detected in the peripheral blood and cerebrospinal fluid of patients with AD [23]. Direct observation has revealed yeast-like cells and hyphal structures in central nervous system (CNS) tissues from AD patients using specific polyclonal antibodies targeting various fungi [38, 39]. This body of evidence conclusively demonstrates the presence of multiple fungal infections in the brains of AD patients. In addition, studies have found that the predominant fungal genera identified in AD patients include *Alternaria*, Botrytis, Candida and Malassezia, while contrasting with Fusarium, which is prevalent in the control group's brains and the composition of fungi in the brain of AD patients is different from that of the control group [40, 41]. At a familial level, *Burkella* is found at higher concentrations in the brains of AD patients than controls [41].

## 4. The Impact of Fungal Toxins and Other Fungal Metabolites on AD

Mycotoxins are potential environmental risk factors for neurodegenerative diseases. These toxins can penetrate the central nervous system through a compromised blood-brain barrier and induce neurotoxicity through multiple mechanisms, such as oxidative stress, inflammation, mitochondrial dysfunction, and the activation of apoptosis and autophagy pathways [16]. Several studies have shown that the impact of these mycotoxins on the nervous system is closely related to the onset and development of neurodegenerative diseases, including AD.

Aflatoxin B1 (AFB1), T-2 toxin, and deoxynivalenol (DON) are capable of crossing the blood-brain barrier, inducing oxidative stress, inflammation, mitochondrial dysfunction, and neuronal apoptosis, thereby damaging neuronal structure and synaptic transmission function [42]. AFB1 not only inhibits the brain's antioxidant system but also induces lipid peroxidation and abnormal enzyme activity (e.g., increased acid/base phosphatase, aspartate aminotransferase, and decreased creatine kinase), with toxicity that is time-dependent. Histological evidence suggests that AFB1 causes brain vascular dilation, neuronal necrosis, and astrocyte proliferation, indicating that it may contribute to the development of neurodegenerative diseases like Alzheimer's [43].

Moreover, trichothecene toxins (such as T-2 toxin) and DON are considered among the most neurotoxic mycotoxins. T-2 toxin triggers neurotoxic reactions by altering mitochondrial function and activating oxidative stress pathways. Specifically, T-2 toxin activates signaling pathways such as Nrf2/HO-1 and p53, leading to mitochondrial dysfunction and causing neuronal damage through processes like reactive oxygen species (ROS) generation, lipid peroxidation, and protein carbonylation. T-2 toxin also increases blood-brain barrier permeability, entering the brain and exacerbating oxidative stress, further contributing to neuronal cell death [42]. DON primarily induces damage to the gut and nervous system through mitochondrial dysfunction and the endoplasmic reticulum-mitochondria calcium pathway (IP3Rs–MCU axis). In pig and intestinal epithelial cell models, DON upregulates Mitofusin 2 expression, increases mitochondrial-endoplasmic reticulum contact areas, and activates the GRP75-VDAC1-IP3Rs complex, leading to calcium overload and oxidative stress, which impair mitochondrial function and epithelial barrier integrity [44].Both AFB1 and T-2 toxin can weaken the blood-brain barrier by damaging brain vascular endothelium or activating astrocytes, forming a vicious cycle of oxidative stress, endoplasmic reticulum stress, inflammation, and mitochondrial damage. Glial cells play a key role in this process, including reducing glutamate buffering capacity, activating the TLR4-Myd88-NF-κB pathway, promoting pro-inflammatory factor release, and disrupting the blood-brain barrier, further exacerbating neurotoxicity [45].

Additionally, Ochratoxin A (OTA) can activate microglia and release pro-inflammatory factors such as IL-1β and IL-18, triggering neuroinflammation [46, 47]. The mycotoxin gliotoxin produced by *Aspergillus fumigatus* selectively induces apoptosis of astrocytes and oligodendrocytes, disrupts the blood-brain barrier, exacerbates oxidative stress, and may trigger neurodegeneration [48]. Common indoor molds like *Aspergillus versicolor* have also been associated with neuroinflammation, with animal inhalation experiments showing their ability to activate glial cells and induce abnormal neuropeptide expression [49].

Furthermore, secretions from *Candida albicans*, such as aspartic proteases (Saps) and candidalysin, can jointly clear brain infections through Saps-Aβ-TLR4 and candidalysin-CD11b mechanisms. When CD11b recognition is blocked, fungal infection duration significantly prolongs, indicating that Candida albicans can clear brain parenchyma via the Saps-Aβ-candidalysin -CD11b-mediated innate immune mechanism [50].

In conclusion, various mycotoxins contribute to neurotoxicity through multiple mechanisms, including altering blood-brain barrier permeability, oxidative stress, inflammation, mitochondrial dysfunction, and abnormal activation of glial cells. These findings suggest their potential etiological role in neurodegenerative diseases.

**Figure 1.**
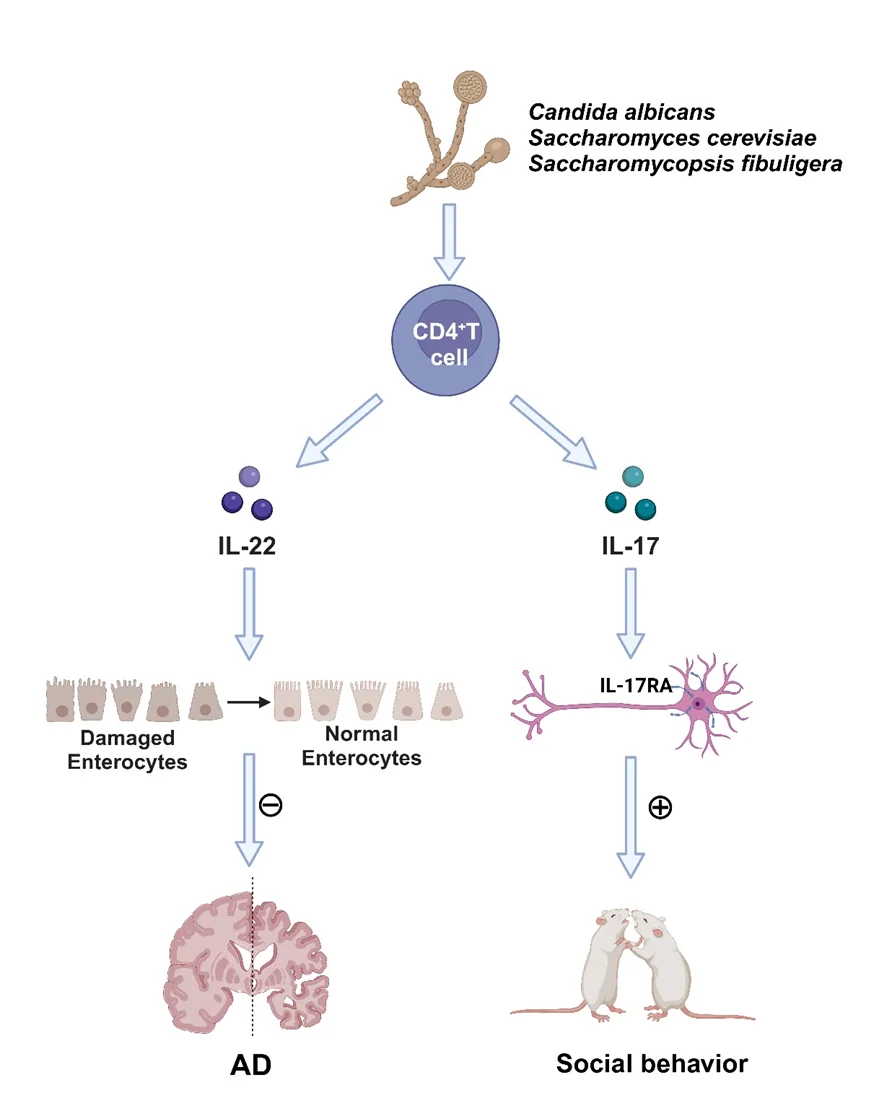
**Immune Modulation by Mucosa-Associated Fungi in AD.** *Candida albicans, Saccharomyces cerevisiae* and *Saccharomycopsis fibuliger*a can promote intestinal mucosal epithelial recovery and social behavior in mice by mediating the secretion of IL-22 and IL-17 by CD4+T cells.

## 5. Mechanisms of Intestinal Fungi Influence on AD

Intestinal fungi contribute to AD progression through distinct mechanisms that incite unique inflammatory responses [48]. Primarily, they exert their influence via two pathways: mediated immunity by mucosa-associated fungi and indirect effects through chito-oligosaccharides (COS).

### 5.1 Immune Modulation by Mucosa-Associated Fungi in AD.

Recent studies have highlighted that IL-22 contributes to maintaining the integrity and functionality of the intestinal epithelium, facilitating epithelial cell proliferation and repair, and fortifying mucosal barrier resistance of the mucosal barrier to prevent the invasion of pathogens [51-53]. Interleukin-17A (IL-17A) also exhibits neuroregulatory effects, significantly impacting the social behaviors of mice [54-57]. Mucosa-associated fungi, including *Candida albicans*, *Saccharomyces cerevisiae*, and *Saccharomycopsis fibuligera* can induce IL-22 through CD4+ Th cells, thereby bolstering intestinal epithelial health and guarding against damage and infection. Furthermore, the interaction between these fungi and IL-17 signaling has been observed to influence social behavior in neuronal models [58]. However, the specific mechanisms through which IL-17 signaling occurs remain to be fully elucidated, and there are doubts about whether these results can be replicated in humans (Fig. 1). Additionally, this research underscores the potential of certain fungi as targets for developing novel AD prevention and treatment strategies.

### 5.2 Chitosan Oligosaccharides (COS) influence AD through multiple pathways

**Figure 2.**
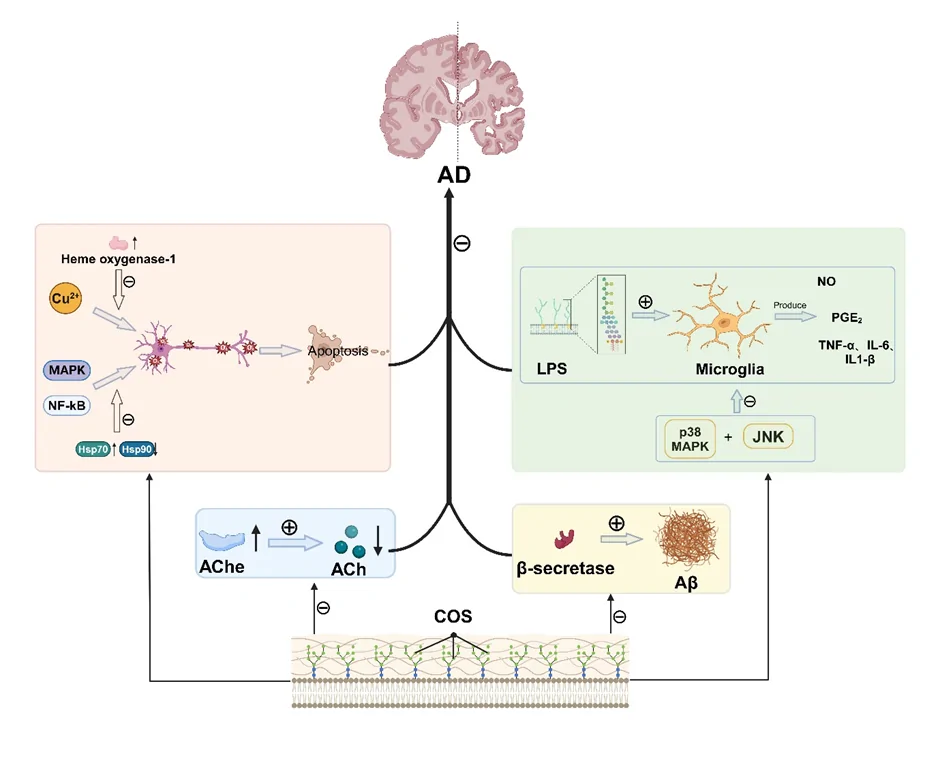
**COS can alleviate Alzheimer's Disease (AD) through multiple pathways.** COS can alleviate AD by inhibiting the production of NO, PEG2, TNF-α, IL-6 and IL-1β, alleviating oxidative stress in neurons, reducing AChE overexpression, and reducing Aβ production.

Chitosan oligosaccharides (COS), significant degradation products of chitin, and chitin is a distinctive component of fungal cell walls [59], play a pivotal role in influencing AD through various pathways. Research indicates that COS can mitigate AD's onset and progression through mechanisms such as anti-neuroinflammation, antioxidation, inhibition of β-amyloid, acetyl-cholinesterase inhibition, and Cu2+ adsorption. The mechanisms are as follows: (1) The chronic inflammatory responses mediated by glial cells (e.g., astrocytes and microglia) play important roles in AD pathogenesis. And COS can inhibit the production of NO, PEG2, TNF-α, IL-6 and IL-1β by LPS-induced microglia by inhibiting JNK and p38MAPK, thus inhibiting the LPS-induced inflammatory response of microglia [60]. (2)COS could reduce the oxidative stress and prevent the subsequent apoptosis in neurons through heat shock response, particularly the up-regulation of heat shock protein 70 (HSP 70) as well as down-regulation of HSP 90 leading to the suppression of the MAPK and NF-κB pathways [61, 62].(3)Substantial alternative in vitro studies revealed that COS with MW 3–5 kDa and DD~90%, can inhibit β-secretase at IC-50 of 25 μM to 42 μM, thereby reducing the deposition of Aβ [63]. (4) Decreased cholinergic neurotransmission due to over-expression of acetylcholinesterase (AChE) is also involved in the pathogenesis of AD [64], and COS has been shown to be an inhibitor of AChE [65, 66], so COS may have a favorable effect on AD by inhibiting over-expression of AchE. (5) COS counters Cu2+-induced oxidative stress and neuronal damage in the pathogenesis of AD [67]. Meanwhile, COS can reduce Cu2+-induced oxidative stress and apoptosis in neurons by promoting the NF-E2-related factor-2-mediated up-regulation of heme oxygenase-1, an anti-oxidative and anti-apoptotic enzyme [68] (Fig. 2). Given these effects, COS emerges as a promising candidate for AD therapeutics.

### 5.3 Fungal Invasion of the Central Nervous System Leading to the Onset of AD

Fungi primarily invade the central nervous system (CNS) through three mechanisms: 1) Transcellular migration, which involves receptor-mediated transport dependent on microbial ligands binding to host receptors (e.g., *Cryptococcus neoformans* binds hyaluronic acid to CD44) and nonspecific absorption-mediated transport (e.g., *Candida albicans* binds Als3 to gp96); 2) Paracellular transport, as seen when *Cryptococcus neoformans* uses urease and *Candida albicans* uses proteases to disrupt tight junctions, allowing them to traverse the blood-brain barrier (BBB) via the intercellular space; and 3) the Trojan horse mechanism, where *Cryptococcus neoformans* and *Candida albicans* are phagocytosed by immune cells, which then transport them across the BBB into the CNS [69]. Once fungi breach the BBB and invade the CNS, they can activate various glial cells, induce inflammatory responses, and mediate AD-related pathological changes by regulating the production of amyloid beta (Aβ).

Studies suggest that the generation of β-amyloid (Aβ) in AD is primarily attributed to excitatory neurons (ExNs) but can also be produced by immune cells such as oligodendrocytes (OLs) [70]. After intravenous injection of a small amount of *Candida albicans* in mice, activated microglial and astrocytic cells aggregate around the clustered fungi, forming fungal-induced granulomas. At the periphery of these granulomas, amyloid precursor protein (APP) accumulates and is cleaved to produce Aβ. Additionally, *C. albicans* can activate the transcription factor NF-κB in the CNS, inducing the expression of pro-inflammatory factors such as IL-1β, IL-6, and TNF, further promoting excessive Aβ synthesis and ultimately leading to cognitive dysfunction in the host [48]. Moreover, *Malassezia* can induce inflammation by activating Th1 and Th17 immune responses, while *Aspergillus* and *Alternaria* may participate in the production of acetylcholinesterase inhibitors. The co-existence of these fungi may synergistically promote the onset and progression of AD, highlighting their potential important role in the pathogenesis of AD [71].

### 5.4 The Amyloid Seeding Hypothesis in the Context of Gut Fungi

Currently, there is relatively little literature regarding the role of gut fungi in the amyloid protein seed hypothesis. However, some studies have found that the Sup35NM prion domain of yeast can promote the aggregation of tau protein in vitro, forming distinct fibrous structures, and can also exacerbate tau pathology in tau transgenic mice [72]. Yeast prions such as Sup35NM can induce pathological aggregation of AD-related proteins, including amyloid-β (Aβ) and tau. In vitro, minimal amounts of Sup35NM fibrils markedly accelerate tau fibrillization, indicating that Sup35NM’s β-sheet-rich structure may serve as a structural template for aberrant tau folding [73]. Animal studies further demonstrate that injecting Sup35NM fibrils into the brains of tau-transgenic mice exacerbates tau pathology, underscoring the potential for cross-species protein seeding [73]. Sup35NM also significantly facilitates Aβ fibrillization in vitro, highlighting its broad catalytic activity in neurodegenerative protein aggregation [74]. Koloteva-Levine et al. reported that prion fibrils have active surface properties capable of catalyzing heterologous protein aggregation, emphasizing the importance of microenvironmental factors in cross-seeding processes [75]. Additionally, the yeast prion Ure2p directly interacts with tau and promotes its aggregation; injection of Ure2p-induced tau fibrils into tauopathy model mice significantly worsens tau pathology and cognitive impairments [76]. Other fungal prions, including *Podospora HET-s* and yeast Rnq1, also display conserved structural characteristics and cross-seeding potentials [77, 78], collectively providing important insights into how fungal-derived prions may influence human neurodegenerative protein aggregation.

## 6. Metabolomic and Proteomic Features of Gut Fungi

The proteome and metabolome of gut fungi play a significant role in AD research. One study analyzed plasma samples from 25 healthy individuals and 68 patients with neurodegenerative diseases (44 Parkinson's disease (PD) patients and 24 AD patients), testing for 19 types of mycotoxins and their metabolites. The results revealed that only Ochratoxin A (OTA), Ochratoxin B (OTB), and Sterigmatocystin (STER) were detected. Among these, only OTA and STER underwent statistical analysis, and the findings showed that the STER levels in the patient group were significantly higher than those in the healthy control group, with male patients showing higher OTA levels than female patients [79].

This suggests that in the future, integrating host omics data may improve the accuracy of early AD diagnosis and assist in monitoring disease progression. This offers new research avenues for early screening and personalized intervention in AD.

## 7. Modulation of the Gut Microbiome as a Strategy for Alleviating Cognitive Dysfunction in AD Patients

AD has emerged as a critical public health challenge worldwide [80], while current therapeutic options remain largely inadequate. In recent years, growing evidence has indicated that targeting the gut microbiota—through approaches such as fecal microbiota transplantation (FMT), dietary interventions [81], and the intake of probiotics or prebiotics [82] —may offer promising new directions for AD therapy. Notably, emerging findings suggest that the gut microbiome, particularly the fungal component (mycobiome), plays a significant role in the onset and progression of AD [83]. Therefore, modulating gut fungi and their metabolites is increasingly recognized as a cutting-edge focus in AD research (Table 2).

**Table 2.** Regulating gut microbiomes can treat AD.

Author, Date	Article type	Disease	study cohort/sample size	Treatment method	Medicine	Transplantation technique	follow-up	Results
**Sun, 2019 [84]**	RCT	AD	mice (n=16)	FMT	fresh fecal solution from mice	intragastrically administered once daily for 4 weeks.	N	cognitive functions improved in mice
**Yu, 2019 [85]**	RCT	CD	mice (n=80)	FMT	fresh fecal solution from mice	intragastrically administered once daily for 2 weeks.	N	improved MWM performance,
**Hazan, 2020 [86]**	Case	AD	AD patient (n=1)	FMT	stool suspension from the patient’s 85-year-old wife	single 300 mL FMT infusion	6 m	cognitive functions improved
**Park, 2021 [87]**	Case	AD	AD patient (n=1)	FMT	stool suspension from a 27-year-old man	Two 60ml FMT infusion 3 months apart	3 m	cognitive functions improved
**Chen, 2023 [88]**	single-arm clinical trial	CI	CI patients (n=5)	FMT	Fecal bacteria capsules	Take 40 fecal bacteria capsules per week for 3 weeks	180 d	Scores improved for those with MCI and did not worsen for those with SCI
**Agahi, 2018 [118]**	RCT	AD	AD patients (n=48)	probiotics	*Lactobacillus fermentum, Lactobacillus plantarum*, and *Bifidobacterium lactis* or *Lactobacillus acidophilus, Bifidobacterium bifidum*, and *Bifidobacterium longum*	one capsule every other day, 12 weeks in total	12 w	No difference in TYM scores between the two groups
**Akbari, 2016 [119]**	RCT	AD	AD patients (n=60)	probiotics	*Lactobacillus acidophilus, Lactobacillus casei, Bifidobacterium bifidum*, and *Lactobacillus fermentum*	Take 200 ml/day probiotic milk	12 w	Significant improvement in the MMSE
**Hwang, 2019 [120]**	RCT	MCI	MCI patients (n=100)	Probiotics & prebiotics	DW2009 (a mixture of fermented soybean powder and Lactobacillus plantarum C29 freeze-dried powder)	800 mg/day	12 w	Significant improvement in cognitive function
**Tamtaji, 2019 [121]**	RCT	AD	AD patients (n=79)	Probiotics & prebiotics	selenium, *Lactobacillus acidophilus, Bifidobacterium bifidum*, and *Bifidobacterium longum*	Selenium (200 μg/day), *Lactobacillus acidophilus, Bifidobacterium bifidum*, and *Bifidobacterium longum* (2 × 109 CFU/day each)	12w	Significant improvement in the MMSE
**Roy Sarkar, 2021 [90]**	RCT	CI	mice (n=24)	fungi probiotics	*S. boulardii CNCM I-745*	90 mg/kg	N	Reversed cognitive decline associated with intestinal dysbiosis in mice.
**Cecarini, 2022 [92]**	RCT	AD	Mice (n=40)	fungi probiotics	*Saccharomyces cerevisiae*	6–7 mL of the experimental drink	N	cognitive functions improved in mice

AD, Alzheimer’s disease; CD, cognitive dysfunction; MWM, Morris water maze; CDI, Clostridioides difficile infection; MMSE, Mini-Mental State Examination; CI, cognitive impairment; MCI, mild cognitive impairment; SCI severe cognitive impairment

### 7.1 Fecal Microbiota Transplantation and Probiotic Interventions

Fecal microbiota transplantation (FMT), which reshapes the gut microbial ecosystem, has been shown to reverse cognitive and behavioral impairments in AD mouse models, while enhancing gut microbial diversity and synaptic plasticity [84, 85]. Clinical case studies also report that FMT may alleviate symptoms in elderly AD patients with recurrent *Clostridium difficile* infections, improving cognitive impairment, activities of daily living, and emotional state [86, 87]. Furthermore, a preliminary clinical study suggested that FMT could stabilize or even improve cognitive performance in patients with mild cognitive impairment (MCI) by modulating gut microbiota and affecting serum metabolomic profiles [88]. However, most current FMT studies focus on bacterial components, often overlooking the potential role of fungi and other microbial groups.

Recently, the role of beneficial fungi in AD intervention has begun to attract attention. The probiotic yeast *Saccharomyces boulardii* has been shown to significantly ameliorate cognitive deficits, Aβ aggregation, synaptic dysfunction, neuroinflammation, intestinal barrier disruption, and fungal dysbiosis in APP/PS1 mice, likely through modulation of the Toll-like receptor (TLR) signaling pathway. These beneficial effects were eliminated following fluconazole treatment, suggesting that the yeast's colonization in the gut is essential for its therapeutic action [89-91]. Additionally, consumption of yeast-rich dietary components such as beer was found to improve learning and memory in 3×Tg-AD mice [92]. These findings suggest a potentially unique role for gut fungi or fungal probiotics in the prevention and treatment of AD.

### 7.2 Neuroprotective Effects of Fungal-Derived Prebiotics

Fungal-derived prebiotics have also shown promise in AD prevention and therapy [93]. For example, mannan oligosaccharides (MOS) exert neuroprotective effects by reshaping the gut microbiota, strengthening intestinal barrier function, and promoting the production of short-chain fatty acids (SCFAs), which in turn suppress neuroinflammation, regulate hypothalamic–pituitary–adrenal (HPA) axis dysfunction, and reduce Aβ accumulation in the cortex, hippocampus, and amygdala [94, 95], Yeast β-glucan, another fungal-derived prebiotic, not only supports SCFA production and microbiota modulation but also alleviates neuro-inflammation and insulin resistance, exerting significant neuroprotective effects in early-stage AD [96]. In addition, chitosan oligosaccharides (COS), derived from fungal cell walls, have drawn attention for their immunomodulatory and anti-inflammatory properties, particularly their potential in inhibiting microglial activation and improving neurodegenerative pathology [97].

### 7.3 Microecological Detoxification Strategies in AD Management

As previously discussed, mycotoxins, secondary metabolites produced by certain fungi, may contribute to AD pathogenesis via mechanisms involving neuro-inflammation and oxidative stress. Thus, developing microbe-based detoxification strategies to remove these toxins could provide a novel avenue for AD prevention. Studies have demonstrated that probiotic strains such as Bacillus licheniformis TR284 can completely eliminate enniatin B (ENB), while TR388 significantly reduces deoxynivalenol (DON) levels by 31.9% [98]. Another strain, WF2020, not only mildly inhibits the growth of Aspergillus flavus but completely blocks the production of aflatoxin B1 (AFB1), potentially by downregulating the expression of key aflatoxin biosynthetic genes such as aflR and aflS [99]. Furthermore, members of the Lactobacillus genus and *Saccharomyces cerevisiae* have demonstrated broad-spectrum mycotoxin-degrading abilities, effectively reducing concentrations of AFB1, fumonisins (FUMs), T-2 toxin, zearalenone (ZEA), and DON, with the most notable degradation observed for FUMs and comparatively weaker effects for DON [100]. These findings support the concept of modulating gut microbiota to reduce the toxic load of harmful fungal metabolites, providing a theoretical foundation for AD intervention strategies.

### 7.4 Dietary Interventions for Gut Fungal Remodeling

As a safe, non-pharmacological approach, dietary interventions also show promise in modulating the gut fungal community. A study involving MCI patients revealed that six weeks of modified Mediterranean ketogenic diet (MMKD) reduced the abundance of AD-related pathogenic fungi such as Saccharomyces and *Claviceps*, while increasing potentially beneficial fungi including *Agaricus* and *Mrakia.* This dietary intervention also enhanced SCFA production, improved gut barrier integrity, and suppressed inflammation, alongside reductions in cerebrospinal fluid levels of Aβ and phosphorylated Tau—suggesting a potential cognitive benefit via the gut-brain axis [21, 101]. In contrast, although conventional antifungal agents can eliminate pathogenic fungi, they may also disrupt microbial homeostasis and promote the development of resistant strains. Therefore, gut fungal modulation via dietary means offers a safer and more sustainable strategy, though its long-term efficacy still warrants further clinical investigation.

In summary, targeting gut fungi presents a novel approach for the prevention and treatment of AD. Strategies such as probiotics, prebiotics, microbial detoxification, and dietary interventions have shown promising potential. However, current research is still in its early stages, and further clinical evidence is needed to confirm their efficacy and safety.

## 8. Discussion

Emerging evidence increasingly implicates the gut mycobiome in AD. As populations age globally, AD becomes an escalating health challenge, prompting investigation into nontraditional pathogenic contributors such as intestinal fungi. Postmortem analyses have identified the presence of specific fungi (e.g., *Alternaria, Botrytis, Candida*, and *Malassezia*) in the brains of AD patients, suggesting a possible fungal contribution to neuroinflammation and pathology [102]. Even in the prodromal stage of mild cognitive impairment (MCI), alterations in gut fungal communities—such as elevated Botrytis and Cladosporium—have been observed [103]. In established AD, fecal mycobiome analyses have reported enrichment of *Candida tropicalis* and *Schizophyllum* commune, alongside decreased *Rhodotorula mucilaginosa* [104]. Animal models of AD, notably 3×Tg-AD mice, similarly demonstrate dysbiosis, with a marked increase in fungi of the *Dipodascaceae* family [105]. These observations underscore gut fungal alterations as potential early indicators or active participants in AD pathogenesis. Currently, the diagnosis of AD primarily relies on biomarkers such as Aβ and tau proteins, detected through cerebrospinal fluid (CSF), blood plasma, or PET-CT imaging. However, these methods present notable limitations: CSF collection is invasive, PET-CT is costly and not widely accessible, and blood-based assays still face challenges in sensitivity and specificity. As such, there is increasing interest in identifying novels, less invasive biomarkers for AD diagnosis.

Several mechanisms have been proposed linking gut fungi with AD pathology. Evidence from animal models suggests gut fungi can translocate to the central nervous system (CNS), eliciting local inflammatory responses. Specifically, peripheral administration of Candida albicans to mice caused aggregation of microglia and astrocytes around fungal lesions, driving amyloid precursor protein (APP) cleavage and subsequent Aβ accumulation [106]. Additionally, Candida albicans activates nuclear factor κB (NF-κB)-mediated cytokine pathways (e.g., IL-1β, IL-6, TNF-α), further exacerbating Aβ pathology and cognitive deficits [106]. Other fungi, such as Malassezia, also stimulate Th1 and Th17 immune responses, potentially collaborating with other fungal pathogens like *Aspergillus* and *Alternaria* to amplify neuroinflammation [107]. Moreover, fungal prion-like proteins (e.g., yeast Sup35NM) have demonstrated cross-species seeding capability, significantly accelerating aggregation of human tau and Aβ in vitro, and intensifying tau pathology in animal models [73, 74]. These interactions suggest fungal products may directly contribute to protein misfolding characteristics of AD.

Beyond direct CNS invasion, gut fungi influence AD indirectly through immune modulation and metabolic pathways. Commensal fungi, such as *Saccharomyces cerevisiae*, stimulate gut-associated CD4⁺ T cells to produce IL-22, enhancing intestinal barrier integrity, potentially reducing systemic inflammation implicated in neurodegeneration [108]. Conversely, dysbiosis may shift host immunity toward pro-inflammatory Th1 responses, aggravating neuroinflammation. Indeed, animal studies show transfer of microbiota from AD mice to healthy controls promotes peripheral Th1 cell expansion and microglial activation, whereas transferring healthy microbiota into AD mice decreases inflammation markers [109]. Fungal-derived metabolites, including chitin oligosaccharides (COS), exhibit neuroprotective effects by attenuating microglial inflammatory signaling and reducing Aβ production [110]. Collectively, these findings highlight gut fungi as modulators of both peripheral and central inflammatory processes associated with AD.

Given these associations, gut fungi represent promising biomarker candidates. Current AD biomarkers—such as cerebrospinal fluid (CSF) Aβ/tau levels or PET imaging—are invasive, costly, or technically demanding. In contrast, fecal fungal analysis is non-invasive, affordable, and scalable. Notably, fungal metabolites have already been detected in AD patient plasma: sterigmatocystin levels were significantly elevated compared to controls, suggesting potential biomarker utility [111]. Other fungal proteins and polysaccharides (enolase, β-tubulin, chitin, and β-glucan) have been identified in AD patient brain tissue, serum, and CSF [112, 113]. Integrating fungal biomarkers into clinical practice could enhance early AD detection and monitoring.

Therapeutically targeting the gut mycobiome offers novel opportunities for AD intervention. Probiotic yeasts, notably Saccharomyces boulardii, have shown efficacy in animal models: oral administration significantly improved cognition, reduced Aβ deposition, inhibited Toll-like receptor-mediated inflammation, and restored fungal community balance in APP/PS1 mice [114]. Dietary interventions enriched with yeast have similarly demonstrated cognitive benefits. Prebiotic strategies utilizing fungal derivatives (mannan and β-glucan oligosaccharides) have reshaped gut microbiota composition, reduced neuroinflammation, and mitigated brain Aβ accumulation [115]. Additionally, probiotic strains like *Lactobacillus* and *Saccharomyces cerevisiae* effectively degrade neurotoxic mycotoxins, potentially lowering toxin-associated AD risks [116]. Preliminary fecal microbiota transplantation (FMT) studies further support cognitive improvements in AD patients through restoration of microbiome equilibrium. Thus, fungal-targeted interventions hold therapeutic promise, though extensive clinical validation remains necessary.

Despite these promising findings, gut fungi research in AD remains preliminary, with significant challenges ahead. Future work should include larger longitudinal cohorts to confirm causal associations and validate fungal biomarkers. Detailed mechanistic studies at cellular and molecular levels are essential to clarify specific fungal impacts on neuronal and glial function. Additionally, standardized fungal sequencing, cultivation, and metabolite analysis methodologies are urgently needed to improve reproducibility. Multi-omics integration (metagenomics, metabolomics, immunomics) could elucidate complex fungus–bacteria interactions in AD pathology. Ultimately, interdisciplinary collaboration among neurologists, microbiologists, immunologists, and bioinformaticians is crucial. As recent reviews emphasize, translating microbiome-targeted therapies into clinical practice requires overcoming numerous knowledge gaps and practical hurdles [117].

In conclusion, emerging evidence strongly positions gut fungi as significant contributors to AD pathogenesis, offering potential as novel diagnostic biomarkers and therapeutic targets. Intensive investigation into fungal roles could reveal transformative strategies for AD prevention, diagnosis, and treatment, significantly impacting public health outcomes in aging populations.

## Data Availability

The data that support the findings of this study are available from the corresponding author upon reasonable request.
